# Effectiveness of BNT162b2 COVID-19 vaccination in prevention of hospitalisations and severe disease in adults with SARS-CoV-2 Delta (B.1.617.2) and Omicron (B.1.1.529) variant between June 2021 and July 2022: A prospective test negative case–control study

**DOI:** 10.1016/j.lanepe.2022.100552

**Published:** 2022-12-07

**Authors:** Anastasia Chatzilena, Catherine Hyams, Rob Challen, Robin Marlow, Jade King, David Adegbite, Jane Kinney, Madeleine Clout, Nick Maskell, Jennifer Oliver, Leon Danon, Adam Finn, Avon CAP Research Group, Anna Morley, Amelia Langdon, Anabella Turner, Anya Mattocks, Bethany Osborne, Charli Grimes, Claire Mitchell, Emma Bridgeman, Emma Scott, Fiona Perkins, Francesca Bayley, Gabriella Ruffino, Gabriella Valentine, Grace Tilzey, Johanna Kellett Wright, Julia Brzezinska, Julie Cloake, Katarina Milutinovic, Kate Helliker, Katie Maughan, Kazminder Fox, Konstantina Minou, Lana Ward, Leah Fleming, Leigh Morrison, Lily Smart, Louise Wright, Lucy Grimwood, Maddalena Bellavia, Marianne Vasquez, Maria Garcia Gonzalez, Milo Jeenes-Flanagan, Natalie Chang, Niall Grace, Nicola Manning, Oliver Griffiths, Pip Croxford, Peter Sequenza, Rajeka Lazarus, Rhian Walters, Robin Marlow, Robyn Heath, Rupert Antico, Sandi Nammuni Arachchge, Seevakumar Suppiah, Taslima Mona, Tawassal Riaz, Vicki Mackay, Zandile Maseko, Zoe Taylor, Zsolt Friedrich, Zsuzsa Szasz-Benczur

**Affiliations:** a Engineering Mathematics, University of Bristol, Bristol, UK; b Bristol Vaccine Centre, Population Health Sciences, University of Bristol, UK; c Academic Respiratory Unit, University of Bristol, UK; d Clinical Research and Imaging Centre, UHBW NHS Trust, Bristol, UK

**Keywords:** COVID-19, SARS-CoV-2, Respiratory infection, Vaccination

## Abstract

**Background:**

Whilst other studies have reported the effectiveness of mRNA vaccination against hospitalisation, including emergency department or intensive care admission, few have assessed effectiveness against other more clinically robust indices of COVID-19 severity.

**Methods:**

A prospective single-centre test-negative design case–control study of adults hospitalised with COVID-19 disease or other acute respiratory disease between 1 June 2021 and 20 July 2022. We assessed VE (vaccine effectiveness) against hospitalisation, length of stay [LOS] >3 days, WHO COVID Score >5 and supplementary oxygen FiO_2_ (fraction inspired oxygen) >28%, conducting regression analyses controlling for age, gender, index of multiple deprivation, Charlson comorbidity index, time, and community infection prevalence.

**Findings:**

935 controls and 546 cases were hospitalised during the Delta period, with 721 controls and 372 cases hospitalised during the Omicron study period. Two-dose BNT162b2 was associated with VE 82.5% [95% confidence interval 76.2%–87.2%] against hospitalisation following Delta infection, 63.3% [26.9–81.8%], 58.5% [24.8–77.3%], and 51.5% [16.7–72.1%] against LOS >3 days, WHO COVID Score >5, and requirement for FiO2 >28% respectively. Three-dose BNT162b2 protection against hospitalisation with Omicron infection was 30.9% [5.9–49.3%], with sensitivity analyses ranging from 28.8–72.6%. Protection against LOS >3 days, WHO COVID Score >5 and requirement for FiO2 >28% was 56.1% [20.6–76.5%], 58.8% [31.2–75.8%], and 41.5% [−0.4–66.3%], respectively. In the UK, BNT162b2 was prioritised for high-risk individuals and those aged >75 years. In the latter group we found a higher estimate of VE against hospitalisation of 47.2% [16.8–66.6%].

**Interpretation:**

BNT162b2 vaccination results in risk reductions for hospitalisation and multiple patient outcomes following Delta and Omicron COVID-19 infection, particularly in older adults. BNT162b2 remains effective against severe SARS-CoV-2 disease.

**Funding:**

AvonCAP is an investigator-led project funded under a collaborative agreement by 10.13039/100004319Pfizer.

**Research in context**

**Evidence before this study**

COVID-19 vaccine effectiveness (VE) is typically measured against infection, hospitalisation, or death, with limited data assessing VE against other outcomes. Large data linkage studies have shown two-dose BNT162b2 VE against hospitalisation of 80–94% and 86–90% against death/invasive mechanical ventilation (IMV) with Delta (B.1.617.2) infection. In contrast, two-dose BNT162b2 VE estimates of 70–98% against hospitalisation with Omicron (B.1.1.529) infection are reported while, three-dose VE estimates are 77–91% (hospitalisation) and 88–97% (death/IMV).

Such studies have also demonstrated waning immune protection occurring as the interval between most recent COVID-19 vaccination and date of SARS-CoV-2 positive test increases, suggesting that the protection against both variants decreases over time.

**Added value of this study**

We provide the first estimates of two- and three-dose BNT162b2 effectiveness against respiratory failure requiring FiO2 >28% and any form of positive pressure ventilatory support. This analysis shows that two-dose BNT162b2 is highly effective in reducing hospitalisation, as well as preventing admission >3-days and respiratory failure requiring oxygen supplementation or positive pressure ventilatory support due to Delta infection. Three-dose BNT162b2 was found to be effective in reducing Omicron infection severity, including in older and other at-risk adults, although the VE point-estimates were lower than those obtained for two doses against Delta.

**Implications of all the available evidence**

BNT162b2 vaccination provides effective protection against hospitalisation due to both Delta and Omicron infection. There are also significant benefits in terms of preventing severe disease and other poor patient outcomes, including death, critical care admission, and respiratory failure which require either positive pressure support or oxygen supplementation. However, the benefit provided by vaccination decreases over time since the most recent vaccine dose and this effect appears to be more pronounced in older adults, and may occur in other at-risk patient groups, while it may also vary according to the number of vaccine doses received and the circulating variants.

The continuing emergence of new variants will modulate the effectiveness of current COVID-19 vaccines. When combined with waning vaccine-induced immunity, careful monitoring of the effectiveness of COVID-19 vaccines in the population and how this may impact healthcare resources is warranted.

## Introduction

Following the emergence of the wild-type SARS-CoV-2 strain, replacement with multiple genomic variants occurred.^1^ The B.1.617.2 (Delta) variant emerged in March 2021 and spread quickly, resulting in a surge of SARS-CoV-2 infections and rapid associated increase in UK COVID-19 hospitalisations.^2^ The emergence of Delta was followed by Omicron (B.1.1.529) variant emergence, first detected in South Africa in November 2021,^3^ and subsequently resulting in a fourth wave of SARS-CoV-2 infection in the UK. By 26th December 2021, approximately 95% of UK SARS-CoV-2 infections were caused by Omicron, rapidly replacing Delta as the main circulating variant.^4^ These variants are both characterized by multiple spike protein mutations which may alter cell entry and enable immune evasion, causing concern that immune responses to the N-terminal and receptor-binding domain would be less effective. Vaccine-induced serum neutralising activity against Delta and Omicron variants^5^^,^^6^ is lower than against previous variants and wild-type virus, and Omicron sublineages BA.2.12.1 and BA.4/5 show further immune escape.^7^

Several COVID-19 vaccines received rapid regulatory authorization based on demonstrated efficacy in clinical trials. The main COVID-19 vaccines used in the UK target the spike protein: BNT162b2 (Cominarty®) and mRNA-1273 (Spikevax®) mRNA vaccines, and ChAdOx1 (Vaxzevria®) a replication-deficient simian adenovirus vector vaccine. Initially vaccine effectiveness (VE) was estimated when the original wild-type and then Alpha variant strains (B.1.1.7, the predominant lineage in the UK from January-May 2021) were in circulation, initially assessing one dose VE in adults ≥80 years^8^, ^9^, ^10^ and then other patient age groups.^9^ As patients became eligible for second doses, real-world VE estimates against hospitalisation due to COVID-19 approximated 52–92%.^10^, ^11^, ^12^ UK estimates of three-dose VE against hospitalisation due to Omicron vary, ranging from 70 to 99%.^13^ Early in 2021, the interval between first and second doses of COVID-19 vaccines was extended to 12 weeks in the UK, to prioritise first dose administration.^14^ The booster vaccination program began in September 2021, was timed at 6-months after completion of the primary course, and prioritised the most vulnerable: all adults aged ≥50 years, and those in clinical risk groups.^15^ Thus, UK COVID-19 VE estimates may differ from those in other countries.

Many studies evaluating VE against hospitalisation and severe COVID-19 disease use linkage of large vaccination and admission databases.^10^^,^^16^^,^^17^ With continuing high incidence of SARS-CoV-2 infections, determining whether hospitalised patients who test positive for SARS-CoV-2 actually have COVID-19 becomes increasingly challenging. Admission thresholds also change over time as healthcare resource pressures change and understanding of disease improves. Collecting detailed clinical data on all admissions permitted us to identify relevant cases accurately and to evaluate disease severity using multiple measures. Accordingly, we undertook a test-negative design (TND) case control study comparing COVID-19 and non-COVID-19 patients with acute lower respiratory tract disease (aLRTD) to evaluate the effectiveness of BNT162b2 against severe disease caused by the Delta and Omicron variants.

## Methods

### Study design and conduct

We conducted a TND case–control study of adults admitted to North Bristol and University Hospitals Bristol and Weston NHS Trusts [AvonCAP: ISRCTN17354061] between 1st June 2021 and 20th July 2022 inclusive. Patients who had signs/symptoms of respiratory infection and were aged ≥18 y (years) on hospitalisation were included.^18^ Eligible cases and controls were identified from the medical admission list, and data collected from medical records using REDCap software.^19^ Data collection methods were identical for cases and controls. Vaccination records for each study participant were obtained from linked hospital and GP records, including vaccination brand and date of administration. Collection of vaccination data was undertaken by individuals blinded to participants’ SARS-CoV-2 test results.^8^ To avoid observer bias, all data collection was undertaken by individuals not involved in analysis and blinded to results.

### Case definition and exclusions

Only patients with ≥2 signs of acute respiratory disease or a confirmed clinical/radiological diagnosis of aLRTD were included.^8^ Cases were defined as patients admitted with aLRTD and a positive admission SARS-CoV-2 test, using the UKHSA diagnostic assay in current use. Delta was initially identified by S-gene positive PCR (SGTP) testing, subsequently replaced on 11th May 2021 by P681R target following validation. Omicron was identified as S-gene negative (SGNP) and then by K417N mutation.^20^ Cases without variant identification results admitted between the 1st June 2021 (when Alpha ceased to be dominant) and 7th November 2021 (date of first confirmed Omicron hospitalisation in Bristol) were inferred to be Delta. Those after 7th February 2022 (last date of confirmed Delta admission) were inferred to be Omicron. SARS-CoV-2 positive admissions without identified variant and SARS-CoV-2 negative admissions between 8th Nov 2021 and 6th Feb 2022 were excluded from analysis.

Patients with symptoms starting >10-days prior to admission were excluded (to reduce the chance of including potentially false negative admission SARS-CoV-2 tests), as were those with a confirmed previous SARS-CoV-2 infection. Repeat positive admission results for SARS-CoV-2 were excluded, so only the first COVID-19 admission for each individual was included. We excluded patients who were partially vaccinated, receiving only one-dose or developing symptoms <7-days after their second vaccination, and patients who had received four-doses on admission to hospital.

Controls had to have aLRTD and a negative SARS-CoV-2 result. Controls could have multiple hospitalisations and be included, provided subsequent admissions were >7-days following previous discharge; however, once a patient became a case, further readmissions were excluded. Controls were classified based on their admission date: those admitted between the 1st June 2021 and 7th November 2021 (date of first confirmed Omicron hospitalisation in Bristol) were classified as hospitalised during the Delta dominance period, and those after 7th February 2022 (last date of proven Delta admission) were classified as occurring during Omicron dominance.

### Exposure definition

We studied VE of two-dose and three-dose BNT162b2 (Pfizer-BioNTech, Comirnaty®) against Delta and Omicron SARS-CoV-2 variants respectively, in adults (≥18 y). We also determined VE by age group: <50 y, ≥50 y and ≥75 y. Individuals were defined as vaccinated if they had received two-dose or three-dose BNT162b2 vaccine, and unvaccinated if they had never received any vaccine. Immunisation with two-dose BNT162b2 was defined as having received two-dose BNT162b2 with >7-days having elapsed between the second-dose and symptom onset (and no third-dose received). Immunisation with three-dose BNT162b2 was defined as having received three-dose BNT162b2 with >7-days having elapsed between the third-dose and symptom onset (and no fourth dose received).

### Outcomes

As a primary outcome, VE against hospital admission with either a clinical or radiological aLRTD diagnosis or aLRTD signs/symptoms was assessed.^8^ To investigate COVID-19 disease severity in SARS-CoV-2 positive hospitalised patients, we considered three additional measures; World Health Organisation (WHO) COVID-19 outcome score >5, maximum oxygen requirement fraction inspired (FiO_2_) >28% (both within the first seven days of hospitalisation), and total hospital length of stay (LOS) >3-days. These outcome measures reflect different operational concerns for hospitals, in particular the oxygen supply, availability of high dependency care, and a measure of overall hospital bed utilisation. The WHO score assesses the level of invasive ventilation required (4 = room air, 5 = oxygen supplementation required, 6 = non-invasive positive pressure ventilation usage, 7–9 requires endotracheal intubation).^21^ Thresholds for outcomes used were determined empirically and have been reported previously.^22^

In a secondary analysis, we assessed VE of two-dose and three-dose BNT162b2 against Delta and Omicron SARS-CoV-2 variants, categorising vaccinated individuals based on the time interval between symptom onset and last vaccination into ≤3-months and >3-months and conducting separate analyses for each category by SARS-CoV-2 variant.

### Statistical analysis

Using a TND, VE against hospitalisation was assessed by comparing the odds of testing positive for specific SARS-CoV-2 variants among vaccinated versus unvaccinated participants. VE was defined as (1–OR) × 100, where OR is the odds ratio of testing positive among vaccinated participants compared with unvaccinated participants using univariable regression (unadjusted vaccine effectiveness). Limiting the sample under study to SARS-CoV2 positive participants, VE was assessed against the three severity outcomes of hospitalisation listed above. This is therefore a comparison of the odds of a severe in-hospital outcome within hospitalised individuals with SARS-CoV-2 between vaccinated and unvaccinated individuals.

Due to disease incidence variation, health system capacity and increasing population immunity occurring through vaccination, SARS-CoV-2 infection changes over time could introduce biases and confound results. To mitigate this, we performed multivariable logistic regression analyses for the four outcomes adjusting for age, gender, Index of Multiple Deprivations (IMD, the official measure of relative deprivation for small areas (or neighbourhoods) in England in 2019) decile rank, Charlson comorbidity index (CCI) [continuous variable], week of admission [integer-valued variable], and community SARS-CoV-2 prevalence [lagged by time interval between infection and hospitalisation, assumed to be 10-days for Delta and 8-days for Omicron]^23^ (Adjusted VE - Table 2, Table 3). As a sensitivity analysis, we matched cases and test-negative controls using 1:2 exact matching by age, CCI category and week of hospitalisation where possible, while adjusting for gender, IMD and community SARS-CoV-2 infection prevalence using conditional logistic regression (matched conditional logistic regression Supplementary Tables S1–S3).

**Table 1:** Baseline characteristics of study cohort.

Characteristic	Delta (B.1.617.2) variant			Omicron (B.1.1.529) variant		
	SARS-CoV-2 Positive (*N* = 546)	SARS-CoV-2 Negative (*N* = 935)	*P*-value	SARS-CoV-2 Positive (*N* = 372)	SARS-CoV-2 Negative (*N* = 721)	*P*-value
Vaccination status			<0.001			0.040
Vaccinated^a^	90 (16%)	608 (65%)		187 (50%)	410 (57%)	
Unvaccinated	456 (84%)	327 (35%)		185 (50%)	311 (43%)	
Age - median years (IQR)	52 (38–72)	77 (59–85)	<0.001	75 (51–85)	75 (56–84)	0.4
Sex			0.053			0.6
Male	298 (55%)	461 (49%)		193 (52%)	362 (50%)	
Female	248 (45%)	474 (51%)		179 (48%)	359 (50%)	
LTCF Resident	17 (3.1%)	62 (6.6%)	0.004	28 (7.5%)	31 (4.3%)	0.033
Ethnicity			<0.001			0.3
White British	283 (52%)	717 (77%)		255 (69%)	510 (71%)	
Other	123 (23%)	66 (7.1%)		43 (12%)	63 (8.8%)	
Unknown	140 (26%)	152 (16%)		74 (20%)	147 (20%)	
Index of multiple deprivation	5 (3–7)	5 (3–8)	<0.001	5 (3–8)	5 (3–8)	>0.9
Unknown	13	13		23	54	
Smoking			<0.001			0.4
Current	28 (5.1%)	99 (11%)		43 (12%)	73 (10%)	
Ex-smoker	161 (29%)	406 (43%)		122 (33%)	275 (38%)	
Non-smoker	288 (53%)	345 (37%)		153 (41%)	275 (38%)	
Unknown	69 (13%)	85 (9.1%)		0	1	
Comorbidity scores						
Rockwood Frailty scale			<0.001			0.5
1–4	334 (61%)	409 (44%)		126 (34%)	260 (36%)	
5–10	212 (39%)	525 (56%)		246 (66%)	461 (64%)	
CCI – median (IQR)	1 (0–4)	4 (2–6)	<0.001	4 (1–5)	4 (2–6)	0.093
Respiratory						
Any	124 (23%)	431 (46%)	<0.001	126 (34%)	335 (46%)	<0.001
COPD	42 (7.7%)	246 (26%)	<0.001	65 (17%)	194 (27%)	<0.001
Asthma	73 (13%)	162 (17%)	0.047	59 (16%)	113 (16%)	>0.9
Other^b^	21 (3.8%)	82 (8.8%)	<0.001	12 (3.2%)	73 (10%)	<0.001
Cardiovascular						
Any	80 (15%)	372 (40%)	<0.001	119 (32%)	266 (37%)	0.11
IHD	39 (7.1%)	120 (13%)	<0.001	49 (13%)	91 (13%)	0.8
AF	20 (3.7%)	184 (20%)	<0.001	60 (16%)	129 (18%)	0.5
CCF	22 (4%)	146 (16%)	<0.001	34 (9.1%)	113 (16%)	0.003
Diabetes						
Any	63 (12%)	175 (19%)	<0.001	69 (19%)	124 (17%)	0.6
Type 1	3 (0.5%)	12 (1.3%)	0.8	5 (1.3%)	10 (1.4%)	>0.9
Type 2	60 (10.9%)	163 (17.4%)		64 (1.7%)	114 (15.8%)	
Neurological						
Dementia	12 (2.2%)	73 (7.8%)	<0.001	26 (7.0%)	45 (6.2%)	0.7
Cognitive impairment	8 (1.5%)	44 (4.7%)	<0.001	20 (5.4%)	18 (2.5%)	0.022
CVA	11 (2.0%)	67 (7.2%)	<0.001	17 (4.6%)	38 (5.3%)	0.7
TIA	12 (2.2%)	49 (5.2%)	0.004	16 (4.3%)	37 (5.1%)	0.7
Other neurological disease^c^	14 (2.6%)	31 (3.3%)	0.5	24 (6.5%)	26 (3.6%)	0.046
Oncology						
Solid organ cancer	22 (4.0%)	101 (11%)	<0.001	29 (7.8%)	66 (9.2%)	0.5
Haematological malignancy	13 (2.4%)	16 (1.7%)	0.4	8 (2.2%)	10 (1.4%)	0.5
Renal disease^d^			<0.001			0.081
None	488 (89%)	696 (74%)		282 (76%)	565 (78%)	
Mild	45 (8.2%)	211 (23%)		68 (18%)	133 (18%)	
Moderate/severe	13 (2.4%)	28 (3.0%)		22 (5.9%)	22 (3.1%)	

Data are N (%) unless otherwise stated.

AF, atrial fibrillation; CCF, congestive cardiac failure; CCI, Charlson Comorbidity Index; COPD, chronic obstructive pulmonary disease; CKD; chronic kidney disease; CVA, cerebrovascular accident; IHD, ischaemic heart disease; IQR, interquartile range; LTCF, long-term care facility; TIA, transient ischaemic attack.

a Vaccinated are individuals who have received two doses and three doses of BNT162b2 for Delta and Omicron variant respectively.

b Includes bronchiectasis, pulmonary fibrosis, and other chronic respiratory conditions.

c Includes Parkinson's disease, Huntingdon's disease, and other chronic neurological conditions.

d Mild is CKD stage 1–3; moderate or severe is CKD stage 4–5, end-stage renal failure, or dialysis dependence.

**Table 2:** Vaccine effectiveness for two doses of BNT162b2 against the delta (B.1.617.2) variant.

Characteristic	All adults			<50 years			≥50 years			≥75 years		
	VE (95% CI)	OR (95% CI)	*P*-value	VE (95% CI)	OR (95% CI)	*P*-value	VE (95% CI)	OR (95% CI)	P-value	VE (95% CI)	OR (95% CI)	*P*-value
**1. Hospitalisation**												

Unadjusted vaccine effectiveness												
Two doses	89.4 (86.2–91.9)	0.106 (0.081–0.138)	<0.001	93.1 (85.3–97.2)	0.069 (0.028–0.147)	<0.001	85.5 (80.5–89.3)	0.145 (0.107–0.195)	<0.001	77.9 (65.8–85.8)>	0.221 (0.142–0.342)	<0.001
Adjusted vaccine effectiveness - Logistic regression model												
Two doses	82.5 (76.2–87.2)	0.175 (0.128–0.238)	<0.001	94.4 (87.3–97.8)	0.056 (0.022–0.127)	<0.001	75.6 (65.2–82.9)	0.244 (0.171–0.348)	<0.001	68.2 (47.6–80.6)	0.318 (0.194–0.524)	<0.001
Age		1.005 (0.994–1.016)	0.4		1.057 (1.028–1.088)	<0.001		0.989 (0.972–1.006)	0.2		0.991 (0.954–1.029)	0.7
Sex (male)		1.268 (0.976–1.648)	0.075		1.279 (0.800–2.046)	0.3		1.115 (0.802–1.551)	0.5		1.042 (0.647–1.678)	0.9
CCI		0.810 (0.734–0.890)	<0.001		0.723 (0.503–1.022)	0.070		0.860 (0.774–0.951)	0.004		0.899 (0.771–1.037)	0.2
IMD		0.973 (0.929–1.020)	0.3		1.008 (0.929–1.095)	0.8		0.964 (0.908–1.023)	0.2		0.900 (0.828–0.977)	0.012
Prevalence		1.001 (1.001-1.002)	<0.001		1.001 (1.000–1.002)	0.017		1.001 (1.001-1.002)	<0.001		1.001 (1.000–1.003)	0.017
Week		1.093 (1.072–1.114)	<0.001		1.036 (1.005–1.069)	0.023		1.131 (1.102–1.161)	<0.001		1.150 (1.108–1.198)	<0.001
**2. Hospital admission length >3 days**												

Unadjusted vaccine effectiveness												
Two doses	1.0 (−71.1–41.4)	0.990 (0.586–1.711)	>0.9	70.4 (−40.6–95.8)	0.296 (0.042–1.406)	0.2	35.4 (−25.3–66.1)	0.646 (0.339–1.253)	0.2	69.2 (1.3–92.1)	0.308 (0.079–0.987)	0.061
Adjusted vaccine effectiveness - Logistic regression model												
Two doses	63.3 (26.9–81.8)	0.367 (0.182–0.731)	0.005	74.3 (−32.4–96.6)	0.257 (0.034–1.324)	0.12	56.1 (4.2–80.0)	0.439 (0.200–0.958)	0.039	75.6 (14.0–94.3)	0.244 (0.057–0.860)	0.038
Age		1.034 (1.012–1.055)	0.002		1.054 (1.017–1.093)	0.005		0.995 (0.955–1.035)	0.8		1.008 (0.911–1.122)	0.9
Sex (male)		1.173 (0.779–1.762)	0.4		1.588 (0.906–2.791)	0.11		0.718 (0.375–1.347)	0.3		3.443 (1.063–12.746)	0.048
CCI		1.034 (0.868–1.247)	0.7		0.788 (0.486–1.251)	0.3		1.247 (0.974–1.660)	0.10		1.089 (0.709–1.741)	0.7
IMD		1.045 (0.971–1.126)	0.2		1.052 (0.954–1.161)	0.3		1.053 (0.939–1.185)	0.4		1.066 (0.864–1.335)	0.6
Week		1.027 (1.000–1.055)	0.050		1.026 (0.992–1.063)	0.14		1.033 (0.988–1.081)	0.2		1.018 (0.918–1.130)	0.7
**3. WHO score >5**												

Unadjusted vaccine effectiveness												
Two doses	21.3 (−27.9–51.0)	0.787 (0.490–1.279)	0.3	45.3 (−153.4–89.4)	0.547 (0.106–2.534)	0.4	51.5 (14.2–72.5)	0.485 (0.275–0.858)	0.012	28.6 (−59.5–68.5)	0.714 (0.315–1.595)	0.4
Adjusted vaccine effectiveness - Logistic regression model												
Two doses	58.5 (24.8–77.3)	0.415 (0.227–0.752)	0.004	41.6 (−200.7–89.8)	0.584 (0.102–3.007)	0.5	50.3 (4.1–74.3)	0.497 (0.257–0.959)	0.037	50.5 (−22.4–80.9)	0.495 (0.191–1.224)	0.14
Age		1.023 (1.004–1.042)	0.016		1.053 (1.017–1.093)	0.005		0.968 (0.936–1.000)	0.051		0.959 (0.892–1.029)	0.2
Sex (male)		1.106 (0.756–1.618)	0.6		1.601 (0.911–2.821)	0.10		0.640 (0.361–1.120)	0.12		0.435 (0.176–1.038)	0.065
CCI		1.022 (0.875–1.203)	0.8		0.912 (0.576–1.456)	0.7		1.209 (0.984–1.522)	0.087		1.688 (1.189–2.530)	0.006
IMD		0.940 (0.877–1.007)	0.077		0.914 (0.827–1.007)	0.072		0.979 (0.885–1.084)	0.7		0.957 (0.817–1.123)	0.6
Week		1.010 (0.985–1.036)	0.4		1.014 (0.979–1.050)	0.4		0.997 (0.958–1.038)	0.9		0.976 (0.911–1.046)	0.5
**4. Supplementary oxygen FiO2 >28%**												

Unadjusted vaccine effectiveness												
Two doses	30.3 (−10.0–56.1)	0.697 (0.439–1.100)	0.12	100 (−1.6 × 10^29^–NA)	0 (NA-1.6 × 10^28^)	>0.9	41.6 (2.5–65.2)	0.584 (0.348–0.975)	0.040	6.7 (−97.7–56.0)	0.933 (0.440–1.977)	0.9
Adjusted vaccine effectiveness - Logistic regression model												
Two doses	51.5 (16.7–72.1)	0.485 (0.279–0.833)	0.009	100.0 (−4.5 × 10^27^–NA)	0.000 (NA-4.5 × 10^25^)	>0.9	31.5 (−22.2–61.7)	0.685 (0.383–1.222)	0.2	20.8 (−79.1–65.5)	0.792 (0.345–1.791)	0.6
Age		1.028 (1.010–1.046)	0.002		1.065 (1.028–1.107)	<0.001		0.985 (0.958–1.012)	0.3		0.920 (0.853–0.986)	0.023
Sex (male)		1.190 (0.839–1.687)	0.3		1.092 (0.622–1.915)	0.8		1.082 (0.673–1.739)	0.7		0.635 (0.281–1.405)	0.3
CCI		0.988 (0.927–1.052)	0.7		0.941 (0.850–1.039)	0.2		1.042 (0.957–1.136)	0.3		1.102 (0.956–1.277)	0.2
IMD		0.903 (0.779–1.044)	0.2		0.812 (0.464–1.291)	0.4		1.005 (0.850–1.189)	>0.9		1.387 (1.037–1.914)	0.035
Week		1.009 (0.986–1.033)	0.4		1.022 (0.988–1.058)	0.2		0.989 (0.956–1.022)	0.5		0.963 (0.904–1.024)	0.2

CI, Confidence Interval; CCI, Charlson comorbidity index; FiO2, Fraction inspired oxygen; IMD, index of multiple deprivation; OR, Odds Ratio; VE, vaccine effectiveness; WHO, World Health Organisation.

The results of Matched Conditional Logistic Regression models are shown in Supplementary Data 2.

**Table 3:** Vaccine effectiveness for three doses of BNT162b2 against the Omicron (B.1.1.529) variant.

Characteristic	All adults			<50 years			≥50 years			≥75 years		
	VE (95% CI)	OR (95% CI)	*P*-value	VE (95% CI)	OR (95% CI)	*P*-value	VE (95% CI)	OR (95% CI)	*P*-value	VE (95% CI)	OR (95% CI)	*P*-value
**1. Hospitalisation**												

Unadjusted vaccine effectiveness												
Three doses	28.4 (7.1–44.8)	0.716 (0.552–0.929)	0.012	32.6 (-24–64.2)	0.674 (0.358–1.240)	0.2	26.5 (0.5–45.7)	0.735 (0.543–0.995)	0.046	30.7 (−2.8–53.1)	0.693 (0.469–1.028)	0.067
Adjusted vaccine effectiveness - Logistic regression model												
Three doses	30.9 (5.9–49.3)	0.691 (0.507–0.941)	0.019	42.6 (−17.4–72.7)	0.574 (0.273–1.174)	0.13	32.0 (3.8–52.0)	0.680 (0.480–0.962)	0.029	47.2 (16.8–66.6)	0.528 (0.334–0.832)	0.006
Age		1.001 (0.990–1.012)	0.9		1.029 (0.991–1.071)	0.14		0.994 (0.977–1.011)	0.5		1.005 (0.971–1.041)	0.8
Sex (male)		1.005 (0.756–1.335)	>0.9		0.565 (0.288–1.086)	0.090		1.136 (0.824–1.569)	0.4		1.388 (0.927–2.085)	0.11
CCI		0.957 (0.877–1.042)	0.3		0.824 (0.544–1.196)	0.3		0.972 (0.887–1.063)	0.5		1.010 (0.904–1.128)	0.9
IMD		1.017 (0.967–1.069)	0.5		1.160 (1.035–1.307)	0.012		0.987 (0.932–1.044)	0.6		0.994 (0.926–1.067)	0.9
Prevalence		1.001 (1.001-1.002)	<0.001		1.001 (1.000–1.002)	0.019		1.001 (1.001-1.002)	<0.001		1.001 (1.001-1.002)	<0.001
Week		0.959 (0.929–0.991)	0.011		0.954 (0.891–1.020)	0.2		0.962 (0.926–0.999)	0.042		0.935 (0.889–0.982)	0.008
**2. Hospital admission length >3 Days**												

Unadjusted vaccine effectiveness												
Three doses	−9.1 (−74.1–31.5)	1.091 (0.685–1.741)	0.7	72.8 (−10.4–96)	0.272 (0.040–1.104)	0.11	21.0 (−39.9–55.9)	0.790 (0.441–1.399)	0.4	9.6 (−93.1–59.1)	0.904 (0.409–1.931)	0.8
Adjusted vaccine effectiveness - Logistic regression model												
Three doses	56.1 (20.6–76.5)	0.439 (0.235–0.794)	0.008	74.4 (−24.2–96.9)	0.256 (0.031–1.242)	0.13	49.8 (3.8–74.7)	0.502 (0.253–0.962)	0.042	19.6 (−76.2–64.8)	0.804 (0.352–1.762)	0.6
Age		1.036 (1.014–1.059)	0.001		1.032 (0.953–1.121)	0.4		1.031 (0.999–1.064)	0.060		1.011 (0.946–1.082)	0.7
Sex (male)		1.438 (0.848–2.441)	0.2		2.548 (0.684–9.903)	0.2		1.195 (0.657–2.174)	0.6		1.247 (0.601–2.585)	0.6
CCI		1.088 (0.923–1.298)	0.3		1.986 (0.926–5.133)	0.094		1.056 (0.891–1.267)	0.5		0.995 (0.819–1.222)	>0.9
IMD		1.085 (0.985–1.196)	0.10		0.939 (0.754–1.156)	0.6		1.149 (1.025–1.292)	0.018		1.145 (0.996–1.324)	0.061
Week		0.932 (0.888–0.977)	0.004		0.917 (0.810–1.024)	0.14		0.927 (0.876–0.978)	0.007		0.949 (0.887–1.014)	0.13
**3. WHO score >5**												

Unadjusted vaccine effectiveness												
Three doses	23.4 (−18.7–50.6)	0.766 (0.494–1.187)	0.2	80.7 (−8.8–99.0)	0.193 (0.010–1.088)	0.13	41.5 (4.0–64.5)	0.585 (0.355–0.960)	0.035	43.3 (−6.7–70.2)	0.567 (0.298–1.067)	0.081
Adjusted vaccine effectiveness - Logistic regression model												
Three doses	58.8 (31.2–75.8)	0.412 (0.242–0.688)	<0.001	81.6 (−21.1–99.1)	0.184 (0.009–1.211)	0.14	54.4 (21.6–73.9)	0.456 (0.261–0.784)	0.005	49.2 (0.2–74.7)	0.508 (0.253–0.998)	0.052
Age		1.026 (1.008–1.045)	0.005		0.971 (0.885–1.059)	0.5		1.018 (0.992–1.045)	0.2		1.005 (0.950–1.063)	0.9
Sex (male)		0.934 (0.579–1.501)	0.8		2.982 (0.727–13.043)	0.13		0.770 (0.461–1.284)	0.3		0.730 (0.385–1.376)	0.3
CCI		1.085 (0.948–1.246)	0.2		1.907 (0.916–4.525)	0.10		1.044 (0.909–1.200)	0.5		0.965 (0.815–1.137)	0.7
IMD		1.052 (0.966–1.146)	0.2		1.258 (0.994–1.642)	0.067		1.037 (0.943–1.140)	0.5		1.095 (0.973–1.236)	0.13
Week		0.969 (0.937–1.001)	0.059		0.968 (0.874–1.060)	0.5		0.960 (0.925–0.995)	0.029		0.909 (0.858–0.958)	<0.001
**4. Supplementary oxygen FiO2 >28%**												

Unadjusted vaccine effectiveness												
Three doses	15.9 (−37.6–48.6)	0.841 (0.514–1.376)	0.5	63.2 (−127.2–98.1)	0.368 (0.019–2.272)	0.4	32.3 (−15.7–60.4)	0.677 (0.396–1.157)	0.2	17.7 (−63.3–57.9)	0.823 (0.421–1.633)	0.6
Adjusted vaccine effectiveness - Logistic regression model												
Three doses	41.5 (−0.4–66.3)	0.585 (0.337–1.004)	0.053	64.6 (−147.5–98.3)	0.354 (0.017–2.475)	0.4	37.1 (−11.2–64.5)	0.629 (0.355–1.112)	0.11	15.6 (−74.7–58.6)	0.844 (0.414–1.747)	0.6
Age		1.036 (1.015–1.058)	<0.001		1.041 (0.938–1.160)	0.4		1.024 (0.996–1.053)	0.10		0.993 (0.936–1.053)	0.8
Sex (male)		0.899 (0.537–1.505)	0.7		2.821 (0.544–15.230)	0.2		0.760 (0.440–1.311)	0.3		0.858 (0.433–1.709)	0.7
CCI		0.919 (0.781–1.067)	0.3		1.548 (0.620–3.352)	0.3		0.900 (0.759–1.050)	0.2		0.829 (0.662–1.010)	0.080
IMD		0.970 (0.882–1.065)	0.5		1.204 (0.916–1.640)	0.2		0.954 (0.860–1.056)	0.4		1.002 (0.881–1.139)	>0.9
Week		0.986 (0.950–1.021)	0.4		0.967 (0.851–1.075)	0.6		0.979 (0.940–1.017)	0.3		0.903 (0.846–0.958)	0.001

CI, Confidence Interval; CCI, Charlson comorbidity index; FiO2, Fraction inspired oxygen; IMD, index of multiple deprivation; OR, Odds Ratio; VE, vaccine effectiveness; WHO, World Health Organisation.

The results of Matched Conditional Logistic Regression models are shown in Supplementary Data 3.

We performed additional subgroup analyses to determine VE in <50 y, ≥50 y and ≥75 y (Table 2, Table 3, Supplementary Tables S2 and S3) for all combinations of outcome and method, and VE by time since last vaccination as ≤3-months, >3-months (Table 4, Supplementary Tables S4 and S5). Further sensitivity analyses were implemented for VE against Omicron to reconfirm that the observed apparent low VE findings were robust. We conducted sensitivity analyses based on matched conditional logistic regression using both stratum matching (relaxing the matching criteria for the exact matching) and distance matching (propensity score; nearest neighbour and genetic matching) methods, and calculated additional adjusted VE estimates using logistic regression models designed to control for time varying-effects of vaccination and incidence by including time as an integer-valued variable, a categorical variable or spline function (Supplementary Table S6).

**Table 4:** Vaccine effectiveness of BNT162b2 against hospitalisation with Delta (B.1.617.2) and Omicron (B.1.1.529) variant by time since last vaccination.

Characteristic	All adults			<50 years			≥50 years			≥75 years		
	VE (95% CI)	OR (95% CI)	*P*-value	VE (95% CI)	OR (95% CI)	*P*-value	VE (95% CI)	OR (95% CI)	*P*-value	VE (95% CI)	OR (95% CI)	*P*-value
**Delta variant**												

Two doses ≤3 months - Unadjusted vaccine effectiveness												
Two doses	96.5 (93.4–98.3)	0.035 (0.017–0.066)	<0.001	94.0 (82.4–98.6)	0.060 (0.014–0.176)	<0.001	96.3 (92.2–98.6)	0.037 (0.014–0.078)	<0.001	97.0 (90.1–99.5)	0.030 (0.005–0.099)	<0.001
Two doses ≤3 months - Adjusted vaccine effectiveness (Logistic regression model)												
Two doses	91.5 (83.1–96.2)	0.085 (0.038–0.169)	<0.001	94.2 (82.1–98.7)	0.058 (0.013–0.179)	<0.001	83.7 (61.8–94.1)	0.163 (0.059–0.382)	<0.001	88.8 (55.5–98.3)	0.112 (0.017–0.445)	0.006
Age		1.012 (0.998–1.027)	0.10		1.053 (1.023–1.085)	<0.001		0.985 (0.962–1.009)	0.2		0.993 (0.936–1.053)	0.8
Sex (male)		1.319 (0.966–1.805)	0.082		1.367 (0.847–2.212)	0.2		1.070 (0.690–1.660)	0.8		1.075 (0.496–2.331)	0.9
CCI		0.703 (0.613–0.801)	<0.001		0.707 (0.485–1.024)	0.066		0.773 (0.661–0.894)	<0.001		0.810 (0.617–1.035)	0.11
IMD		0.994 (0.940–1.052)	0.8		0.983 (0.904–1.070)	0.7		1.027 (0.949–1.112)	0.5		0.922 (0.806–1.051)	0.2
Prevalence		1.002 (1.001-1.002)	<0.001		1.001 (1.000–1.002)	0.016		1.002 (1.001-1.003)	<0.001		1.002 (1.000–1.004)	0.027
Week		1.075 (1.052–1.099)	<0.001		1.036 (1.005–1.070)	0.024		1.116 (1.080–1.155)	<0.001		1.100 (1.042–1.166)	<0.001
Two doses >3 months - Unadjusted vaccine effectiveness												
Two doses	86.3 (82.0–89.7)	0.137 (0.103–0.180)	<0.001	92.3 (79.6–97.8)	0.077 (0.022–0.204)	<0.001	81.2 (74.5–86.3)	0.188 (0.137–0.255)	<0.001	71.4 (55.3–81.7)	0.286 (0.183–0.447)	<0.001
Two doses >3 months - Adjusted vaccine effectiveness (Logistic regression model)												
Two doses	79.5 (71.5–85.3)	0.205 (0.147–0.285)	<0.001	94.6 (84.4–98.5)	0.054 (0.015–0.156)	<0.001	74.6 (63.4–82.5)	0.254 (0.175–0.366)	<0.001	66.8 (45.4–79.8)	0.332 (0.202–0.546)	<0.001
Age		1.004 (0.992–1.015)	0.5		1.062 (1.031–1.094)	<0.001		0.989 (0.972–1.007)	0.2		0.987 (0.949–1.025)	0.5
Sex (male)		1.301 (0.997–1.700)	0.053		1.301 (0.804–2.107)	0.3		1.148 (0.822–1.605)	0.4		1.030 (0.637–1.666)	>0.9
CCI		0.806 (0.729–0.887)	<0.001		0.672 (0.462–0.959)	0.031		0.864 (0.776–0.956)	0.006		0.896 (0.767–1.036)	0.2
IMD		0.974 (0.929–1.022)	0.3		1.015 (0.934–1.105)	0.7		0.961 (0.904–1.020)	0.2		0.898 (0.826–0.975)	0.011
Prevalence		1.001 (1.001-1.002)	<0.001		1.001 (1.000–1.003)	0.009		1.001 (1.000–1.002)	0.003		1.001 (1.000–1.002)	0.051
Week		1.090 (1.069–1.112)	<0.001		1.034 (1.002–1.068)	0.038		1.125 (1.095–1.156)	<0.001		1.138 (1.093–1.187)	<0.001

**Omicron variant**												

Three doses ≤ 3 months - Unadjusted vaccine effectiveness												
Three doses	−49.8 (−130.8–2.8)	1.498 (0.972–2.308)	0.066	−85.4 (−385.8–27.5)	1.854 (0.725–4.858)	0.2	−44.2 (-136–12)	1.442 (0.880–2.360)	0.14	−64.8 (−206.1–10.8)	1.648 (0.892–3.061)	0.11
Three doses ≤ 3 months - Adjusted vaccine effectiveness (Logistic regression model)												
Three doses	31.0 (−15.3–59.1)	0.690 (0.409–1.153)	0.2	13.3 (−158.2–71)	0.867 (0.290–2.582)	0.8	41.1 (−7.4–68.2)	0.589 (0.318–1.074)	0.087	50.8 (−12.2–79.2)	0.492 (0.208–1.122)	0.10
Age		1.002 (0.988–1.016)	0.8		1.034 (0.990–1.081)	0.13		0.999 (0.975–1.023)	>0.9		1.026 (0.970–1.086)	0.4
Sex (male)		0.953 (0.650–1.398)	0.8		0.586 (0.284–1.189)	0.14		1.197 (0.742–1.935)	0.5		1.584 (0.811–3.115)	0.2
CCI		0.972 (0.856–1.102)	0.7		0.756 (0.469–1.146)	0.2		1.016 (0.885–1.167)	0.8		1.092 (0.904–1.334)	0.4
IMD		1.065 (0.995–1.141)	0.069		1.179 (1.042–1.341)	0.010		1.018 (0.935–1.108)	0.7		1.036 (0.916–1.173)	0.6
Prevalence		1.002 (1.001-1.002)	<0.001		1.001 (1.000–1.003)	0.026		1.002 (1.001-1.002)	<0.001		1.002 (1.001-1.003)	<0.001
Week		0.955 (0.915–0.995)	0.030		0.958 (0.890–1.030)	0.3		0.956 (0.907–1.007)	0.094		0.931 (0.858–1.007)	0.079
Three doses >3 months - Unadjusted vaccine effectiveness												
Three doses	40.4 (21.3–55.0)	0.596 (0.450–0.787)	<0.001	62.1 (18.7–83.8)	0.379 (0.162–0.813)	0.017	36.6 (12.9–53.9)	0.634 (0.461–0.871)	0.005	41.7 (12.3–61.2)	0.583 (0.388–0.877)	0.009
Three doses >3 months - Adjusted vaccine effectiveness (Logistic regression model)												
Three doses	33.9 (8.4–52.4)	0.661 (0.476–0.916)	0.013	57.0 (−4.9–83.6)	0.430 (0.164–1.049)	0.072	31.7 (2.4–52.3)	0.683 (0.477–0.976)	0.036	46.9 (16.3–66.4)	0.531 (0.336–0.837)	0.006
Age		1.002 (0.991–1.014)	0.7		1.026 (0.987–1.068)	0.2		0.994 (0.976–1.012)	0.5		1.008 (0.972–1.045)	0.7
Sex (male)		1.010 (0.750–1.360)	>0.9		0.656 (0.326–1.294)	0.2		1.097 (0.782–1.539)	0.6		1.406 (0.921–2.158)	0.12
CCI		0.955 (0.868–1.047)	0.3		0.867 (0.576–1.253)	0.5		0.967 (0.875–1.067)	0.5		0.987 (0.876–1.109)	0.8
IMD		1.016 (0.964–1.071)	0.5		1.159 (1.030–1.309)	0.016		0.987 (0.929–1.048)	0.7		0.995 (0.924–1.073)	>0.9
Prevalence		1.001 (1.001-1.002)	<0.001		1.001 (1.000–1.002)	0.2		1.001 (1.001-1.002)	<0.001		1.001 (1.001-1.002)	<0.001
Week		0.970 (0.937–1.004)	0.084		0.944 (0.878–1.011)	0.10		0.980 (0.941–1.021)	0.3		0.961 (0.911–1.014)	0.15

CI, Confidence Interval; CCI, Charlson comorbidity index; FiO2, Fraction inspired oxygen; IMD, index of multiple deprivation; OR, Odds Ratio; VE, vaccine effectiveness; WHO, World Health Organisation.

The results of Matched Conditional Logistic Regression models are shown in Supplementary Data 4.

Basic characteristics of the sample under study were compared using Fisher exact tests (categorical variables) and Wilcoxon rank-sum tests (continuous variables). Statistical analyses were performed with R v4.0.2. Missing data were limited to the IMD variable and accounted for <2% during Delta and <6% during Omicron time-periods; no imputation was performed, and all analyses only included participants with complete data. Statistical significance was defined using a 2-sided significance level of α = 0.05.

### Ethics and permissions

The Health Research Authority Research Ethics Committee (East of England, Essex), REC20/EE/0157 approved this study, including use of Section 251 of the 2006 NHS Act under Confidentiality Advisory Group authorisation.

### Role of the funding source

This study was conducted as a collaboration between The 10.13039/100012366University of Bristol (study sponsor) and 10.13039/100004319Pfizer (study funder). The study funder did not undertake any data collection; they collaborated in study design, data analysis and manuscript preparation.

## Results

**Fig. 1:**
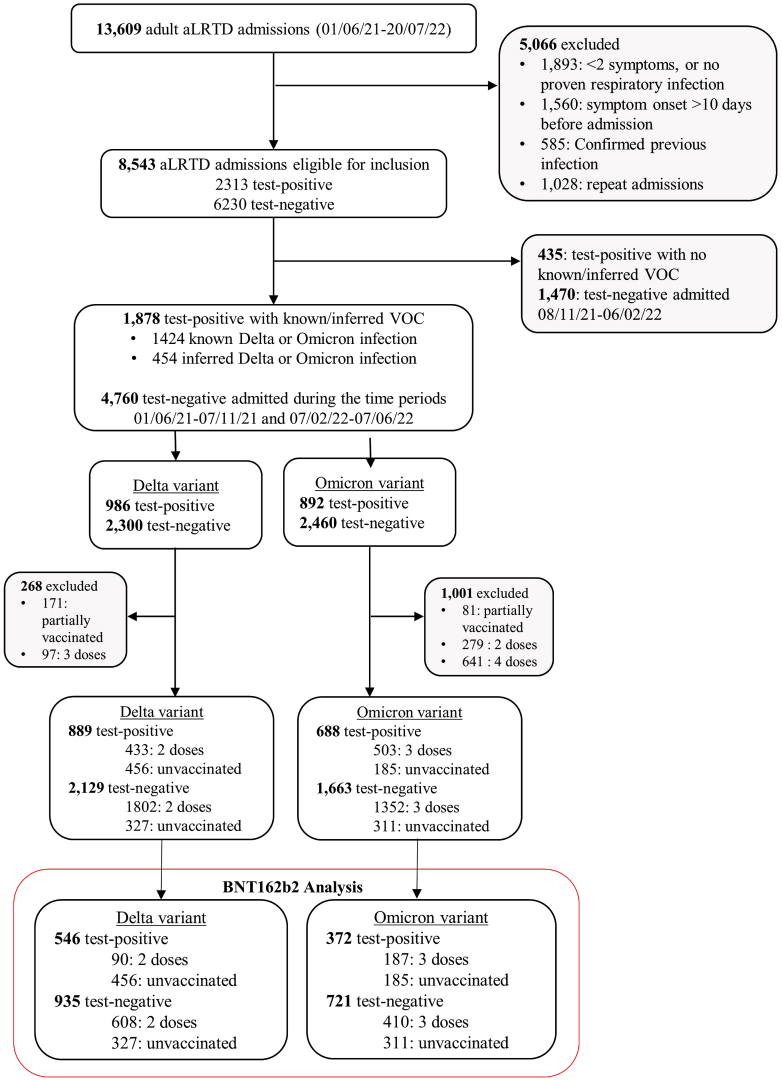
**Study flow diagram.** Inclusion and exclusion criteria in the cohort stratified by SARS-CoV-2 variant and restricted to BNT162b2 vaccine. Of the 546 SARS-CoV-2 positive individuals with known/inferred Delta variant, 90 were vaccinated with two-dose BNT162b2, 456 unvaccinated cases. There were 372 SARS-CoV-2 positive individuals with known/inferred Omicron variant: 182 vaccinated with three-dose BNT162b2, and 185 unvaccinated cases. Of the 935 SARS-CoV-2 negative individuals admitted during the Delta time period, 608 were vaccinated with two-dose BNT162b2 and 327 were unvaccinated cases. In the Omicron time period, 721 SARS-CoV-2 negative individuals were admitted: 410 vaccinated with three-dose BNT162b2 and 311 unvaccinated patients. ∗ During the Delta period, the controls include 51 individuals with 2 respiratory hospitalisations; 6 individuals who were admitted with three separate respiratory admissions, and one individual with 4 separate respiratory admissions. 93.8% of the controls (877/935) had only one respiratory hospitalisation. † In the Omicron period, the controls include 94 individuals with two respiratory admissions, 30 individuals with three separate respiratory admissions, 8 individuals with four separate respiratory admissions and 4 individuals with more than four separate respiratory admissions. 81.1% of the controls (585/721) had only one respiratory hospitalisation. Details of adults hospitalised with SARS-CoV-2 infection are provided in Supplementary Fig. S1. VOC, variant of concern.

During the study period 13,609 adult aLRTD hospitalisations occurred in Bristol, UK: 8543 admissions were eligible for this analysis, with 2313 (27%) testing SARS-CoV-2 positive. Cases and controls designation and exclusions are shown in Fig. 1 and Supplementary Fig. S1. During Delta variant dominance, 698 individuals received two-dose BNT162b2 vaccination >7-days before symptom onset. Among Delta cases, median patient age was 52 y (IQR38-72), 298 individuals (55%) were male, median CCI was 1 (IQR0-4) and patients hospitalised with SARS-CoV-2 aLRTD were significantly younger than those with SARS-CoV-2 negative aLRTD (median age 77 y (IQR59-85), *P* < 0.001). During Omicron variant dominance, 597 individuals had received three-dose BNT162b2 vaccination >7-days before symptom onset. For Omicron cases, median patient age was 75 y (IQR51- 85), 193 (52%) were male, and median CCI was 4 (IQR1-5). The characteristics of cases and controls are listed in Table 1.

Of the 546 Delta SARS-CoV-2 infection cases, 90 (16%) received two-dose BNT162b2, as had 608 of 935 controls (65%); giving an unadjusted VE of 89.4% [95% confidence interval (95%CI) 86–92%]. Adjusted results from logistic regression were VE 82.5% [95%CI 76–87%] and from matched conditional logistic regression were VE 73.6% [95%CI 38–89%]. We found no evidence of a significant trend in VE against hospitalisation with respect to patients’ age (Table 2), which we examined further in the context of an adjusted logistic regression by including additional interaction terms between vaccine and age, and found no evidence that there is a decrease in VE with increasing age (Supplementary Table S7). Two-dose BNT162b2 adjusted VE against disease severity outcomes following Delta infection were 63.3% [95%CI 27–82%], 58.5 [95%CI 25–77%], and 51.5% [95%CI 17–72%] for hospital LOS>3-days, WHO COVID Score >5 and the requirement for FiO2 >28%, respectively (Table 2, Supplementary Table S2).

The effectiveness of three-dose BNT162b2 against hospitalisation with the Omicron variant is shown in Table 3, with an unadjusted VE 28.4% [95%CI 7–45%], and adjusted VE 30.9% [95%CI 6–50%]. Point estimates of VE against hospitalisation showed no consistent trend with age (Table 2). Adjusted VE of three-dose BNT162b2 against hospital LOS >3-days, WHO COVID Score >5 and the requirement for FiO2 >28% was estimated at 56.1% [95%CI 21–77%], 58.8% [95%CI 31–76%], and 41.5% [95%CI 0–66%], respectively.

Sensitivity analyses of three-dose VE against Omicron hospitalisation introduced additional uncertainty, with VE estimates using matched conditional logistic regression ranging from 34.1% [95%CI 0.1–56.6%] to 72.6% [95%CI 19.6–90.7%], and using adjusted logistic regression with alternative time models ranging from 28.8% [95%CI –0.5–49.6%] to 30.9% [95%CI 5.9–49.3%] (Supplementary Table S6). The matched estimates have wider confidence limits resulting from smaller sample sizes, and confidence intervals all overlap.

As protection from vaccination may wane over time, we considered whether VE against hospitalisation and in-hospital outcomes was different when the cohort was stratified by length of time between symptom onset and last COVID-19 vaccination. Adjusted VE against hospitalisation with Delta variant infection of two-dose BNT162b2 administered ≤3-months prior to admission was found to be 91.5% [95%CI 83–96%], and to be 79.5% [95%CI 72–85%] for cases where BNT162b2 had been administered >3-months prior to admission (Table 4). Differences in VE between shorter and longer time since vaccination were larger among older patients, suggesting that waning may occur earlier or be more pronounced with increasing age. Adjusted VE against hospitalisation with Omicron for three-dose BNT162b2 was 31.0% [95%CI −15–59%, *P*-value = 0.2] when administered ≤3-months and 33.9% [95%CI 8–52%, *P*-value = 0.013] when administered >3-months prior to admission. The low numbers of Omicron admissions in the 3-month period following the booster programme rollout resulted in insufficient statistical power to draw firm conclusions about waning following Omicron (Table 4, Supplementary Table S4). VE analyses by time since vaccination against other patient outcomes were also inconclusive due to small case numbers (Supplementary Table S5).

## Discussion

This real-world study provides evidence that BNT162b2 provides protection against hospitalisation from both Delta (B.1.617.2) and Omicron (B.1.1.529) SARS-CoV-2 variants. Whilst other studies have reported mRNA VE against hospitalisation, including emergency department or intensive care admission,^24^ few have assessed effectiveness against other clinically robust COVID-19 severity indices. Our study provides this information, confirming that two-dose BNT162b2 is highly effective in reducing hospitalisation, admission >3-days and respiratory failure requiring oxygen supplementation or positive pressure ventilatory support due to Delta. Further, we find that three-dose BNT162b2 is effective in reducing the risk of admission >3-days, respiratory failure requiring oxygen or WHO outcome score >5 in Omicron infections. This analysis also provides encouraging evidence that BNT162b2 remains effective in reducing admissions against both SARS-CoV-2 variants in patients >75 y: two-dose VE vs Delta: 68.2% [95%CI 47.6–80.6%], and three-dose VE against Omicron: 47.2% [95%CI 16.8–66.6%].

Our analysis estimates two- and three-dose BNT162b2 VE against hospitalisation in the context of the UK COVID-19 vaccine programme.^14^^,^^15^^,^^25^ The estimated two-dose BNT162b2 effectiveness against Delta hospitalisation of 82.5% is similar to other published results (86.7–98.4%).^10^^,^^26^ Our analysis estimated three-dose BNT162b2 VE against Omicron hospitalisation at 30.9% [95%CI 6–50%] for all adults: notably lower than other estimates (varying from 70 to 98%).^13^^,^^24^^,^^27^^,^^28^ Sensitivity analysis with a range of different methods produced comparable estimates with greater or lesser degrees of precision and overlapping confidence intervals. Further, stratified analyses by age found VE counter-intuitively higher in >75 y (for whom point estimate VE against Omicron hospitalisation was 47.2%). A number of potential factors could explain these lower estimates. In the UK, due to the nature of the vaccination programme, three-dose BNT162b2 was only routinely offered to elderly and other high risk groups, who were vaccinated early in the programme.^15^ Linked to this, our estimate may reflect VE after a prolonged period of waning not previously reported. This study has relatively long follow-up and lower Omicron admission numbers in the time-period close to the booster campaign, with 74.3% of vaccinated individuals receiving their third dose >3-months before admission. Additionally, in the UK Omicron variants BA.1/BA.2 were replaced by BA.4/5 in the latter stages of our study period; there is mounting evidence that BA.4/5 show further immune escape beyond that observed for BA.1/BA.2,^7^ which would likely result in lower VE. 10.4% of our study population were admitted during a period when BA.4/5 rates accounted for >50% of UK cases (11th June 2022 and after). Another possibility is that baseline risk of COVID-19 admission reduces over time, as unvaccinated populations acquire immunity following first and any recurrent SARS-CoV-2 infection. Since VE is measured against this baseline, decreases in baseline risk result in an apparent VE decrease. This is reflective of the broader epidemiological and immunological context of the pandemic in the UK and is a potential factor we are unable to control for without more detailed information on immunity induced by infection in our study population. Routine testing for anti-nucleocapsid antibodies was not conducted, and we therefore do not have these data available for determining previous infection status of hospitalised individuals within the Bristol area. The counterintuitive finding that VE seemed to be higher in older patients in this study may also be explained by more prevalent infection-induced immunity in younger individuals.

COVID-19 VE is typically measured against infection, hospitalisation or death, with limited data assessing VE against other outcomes. Two or three-dose mRNA vaccine effectiveness to prevent progression to invasive mechanical ventilation or death has been reported as 76% for Alpha, 44% for Delta, and 46% for Omicron variants.^29^ Stowe and colleagues report that three-dose VE against Omicron-hospitalisation requiring oxygen varies from 80 to 94% in 18–64 y to 90–96% in >65 y.^13^ We found two-dose BNT162b2 VE to prevent oxygen supplementation FiO2 >28% and to prevent positive pressure ventilation (WHO score >5) following Delta hospitalisation was 52% and 59%, respectively, while three-dose BNT162b2 showed VE of 59% against Omicron hospitalisation ventilation. Subtle differences in the definition of the outcome measured may account for the variability in point VE estimates. Taken in combination, these results provide reassuring and robust evidence that vaccines reduce hospitalisation risk with COVID-19 and also prevent respiratory failure requiring significant support.

This analysis found some evidence of waning immunity following two-dose BNT162b2 against Delta infection, with the effect most pronounced in older adults. Whilst we report highly detailed data from two hospitals, this study design lacks the large participant numbers available in some other data-linkage studies, which show VE waning against Omicron and Delta SARS-CoV-2 infection.^30^, ^31^, ^32^

Our analysis has several strengths. All COVID-19 vaccinations are available in the UK solely through the NHS without cost or requirement for insurance at the point of delivery. Therefore, the ability to pay for healthcare does not limit vaccine availability, and vaccinated adults are less likely to be wealthier compared to unvaccinated adults than in fee-based or insurance-based health systems. We adjusted for community SARS-CoV-2 prevalence at the time of infection, as a proxy measure of COVID-19 exposure intensity. We also controlled for age and CCI, two characteristics associated with increased risk of severe SARS-CoV-2 infection, as well as likelihood of vaccine receipt. We utilised symptom onset date to define illness start time and report VE by time following vaccination. We are therefore able to define illness onset relative to both most recent COVID-19 vaccination and hospitalisation date accurately, without relying on positive COVID-19 test date (which may vary widely) thus eliminating this source of bias or misclassification. We determined VE estimates against clinically important outcomes which impact healthcare resource planning, such as oxygen requirement. These study results are not dependent on clinical electronic datasets, which may be subject to biases inherent in systems such as ICD-10 coding, as the data used were collected specifically for the study.

Limitations and advantages of TND studies are previously described,^8^^,^^24^^,^^31^ but our current analysis has additional limitations. This study does not measure BNT162b2 effectiveness in individuals who were not hospitalised with COVID-19 or who were asymptomatic, and we cannot determine VE against asymptomatic disease or transmission. There may be treatment bias as certain groups are not hospitalised, patients may die before admission, or otherwise not be referred to hospital. Weak or moderate protection induced by vaccination could also result in slower disease progression with a longer interval from disease onset to hospitalisation for vaccinated individuals - this may result both in false positive and negative PCR results which may have rendered VE estimates imprecise. We used CCI as a proxy for measuring overall comorbidity as it was not feasible to control for each condition in the CCI separately. Since vaccinated people are likely to be less healthy on average, this may result in VE being under-estimated in this analysis. Although the Bristol population is representative of the UK, this cohort was predominantly Caucasian and the studied vaccines may have different effectiveness in individuals from other ethnic backgrounds. Individuals in this study were vaccinated in the UK vaccine programme, in which intervals between COVID-19 vaccine doses were 12-weeks between first and second dose and for the third-dose at least 6-months after completion of the primary series and this may affect the immune protection. We were unable to obtain results that were statistically significant for some analyses due to lack of power and are unable to comment on the relative effectiveness of different combinations of COVID-19 vaccine brands at this time. Additional research using a larger patient cohort may address these limitations and confirm our findings.

In this prospective study, we provide evidence that BNT162b2 remains effective against SARS-CoV-2 infection: including both two-doses against Delta hospitalisation and three-doses against Omicron hospitalisation. Overall, the results are encouraging, but highlight the need for careful consideration of the timing and implementation of additional COVID-19 vaccine doses and healthcare resource planning in the context of ongoing hospitalisation with SARS-CoV-2 infection.

## Contributors

AC, CH, RC, RM, LD, JO, and AF generated the research questions and analysis plan. CH, JK, JK, MC, DA and The Avon CAP team were involved in data collection. AC, CH, RM, RC, LD, and AF undertook data analysis. All authors (AC, CH, RC, RM, JK, DA, JK, MC, NM, JO, LD, AF) were involved in the final manuscript preparation and its revisions before publication. The data was verified by CH, DA, MC, JK and JK. AF provided oversight of the research.

## Data sharing statement

The data used in this study are sensitive and cannot be made publicly available without breaching patient confidentiality rules. Neither the data dictionary nor analysis code are therefore available.

## The AvonCAP research group

Anna Morley, Amelia Langdon, Anabella Turner, Anya Mattocks, Bethany Osborne, Charli Grimes, Claire Mitchell, Emma Bridgeman, Emma Scott, Fiona Perkins, Francesca Bayley, Gabriella Ruffino, Gabriella Valentine, Grace Tilzey, Johanna Kellett Wright, Julia Brzezinska, Julie Cloake, Katarina Milutinovic, Kate Helliker, Katie Maughan, Kazminder Fox, Konstantina Minou, Lana Ward, Leah Fleming, Leigh Morrison, Lily Smart, Louise Wright, Lucy Grimwood, Maddalena Bellavia, Marianne Vasquez, Maria Garcia Gonzalez, Milo Jeenes-Flanagan, Natalie Chang, Niall Grace, Nicola Manning, Oliver Griffiths, Pip Croxford, Peter Sequenza, Rajeka Lazarus, Rhian Walters, Robin Marlow, Robyn Heath, Rupert Antico, Sandi Nammuni Arachchge, Seevakumar Suppiah, Taslima Mona, Tawassal Riaz, Vicki Mackay, Zandile Maseko, Zoe Taylor, Zsolt Friedrich, Zsuzsa Szasz-Benczur.

## Declaration of interests

CH is Principal Investigator of the AvonCAP study which is an investigator-led University of Bristol study funded by 10.13039/100004319Pfizer and has previously received support from the NIHR in an Academic Clinical Fellowship. JO and LD are Co-Investigators on the AvonCAP Study. AF is a member of the Joint Committee on Vaccination and Immunization (JCVI) and chair of the World Health Organization European Technical Advisory Group of Experts on Immunization (ETAGE) committee. In addition to receiving funding from 10.13039/100004319Pfizer as Chief Investigator of this study, he leads another project investigating transmission of respiratory bacteria in families jointly funded by 10.13039/100004319Pfizer and the Gates Foundation. The other authors have no relevant conflicts of interest to declare.

## Figures and Tables

**Figure:**
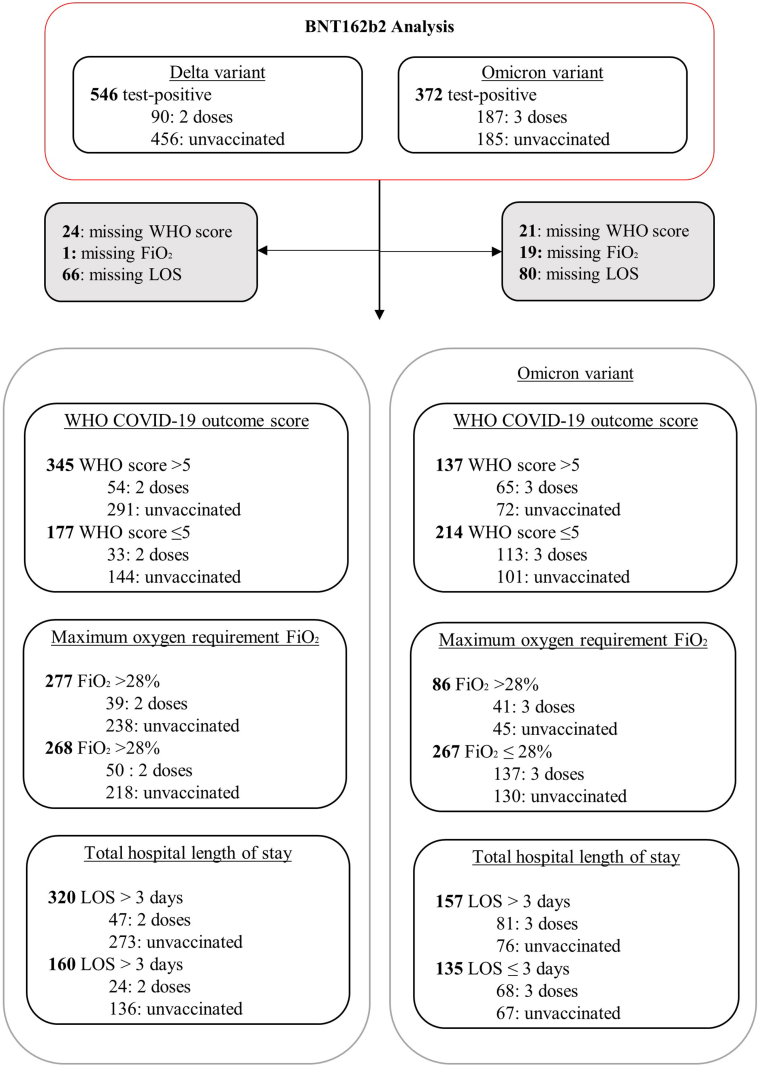
Supplementary Fig. S1

## Appendix A Supplementary data

Supplementary Tables S1–S7

Caption for Supplementary material

Chatzilena STROBE checklist
