# Supplementary material for: Effectiveness of BNT162b2 COVID-19 vaccination in prevention of hospitalisations and severe disease in adults with SARS-CoV-2 Delta (B.1.617.2) and Omicron (B.1.1.529) variant between June 2021 and July 2022: A prospective test negative case–control study
Source: Lancet Reg Health Eur. 2022 Dec 7;25:100552. doi: 10.1016/j.lanepe.2022.100552 (PMC9728025; doi:10.1016/j.lanepe.2022.100552)
Supplement: Caption for Supplementary material [file mmc2.docx]

**Effectiveness of BNT162b2 COVID-19 vaccination in prevention of hospitalisations and severe disease in adults with Delta (B.1.617.2) and Omicron (B.1.1.529) variant SARS-CoV-2 infection: a prospective test negative case-control study**

**Supplementary Information**

**Supplementary Figure 1: Flow diagram of adults hospitalised with SARS-CoV-2 infection.**

Missing data excluded for each severity outcome, stratified by SARS-CoV-2 variant.

**Supplementary Table 1: Factors used in matched conditional logistic regression analyses (Supplementary Tables 2-3)**

Charlson comorbidity index (CCI) category: None, CCI=0; Mild, CCI=1-2; Moderate; CCI=3-4; Severe, CCI >4

**Supplementary Table 2: Vaccine effectiveness for two-dose BNT162b2 against the Delta (B.1.617.2) variant**

CI, Confidence Interval; CCI, Charlson comorbidity index; FiO2, Fraction inspired oxygen; IMD, index of multiple deprivation; OR, Odds Ratio; VE, vaccine effectiveness; WHO, World Health Organisation

*All adults: 85 test-positive cases were matched to 95 test-negative controls with no match found for 448 cases and 827 controls
(matched by age, CCI category and week of admission).
<50y: 109 test-positive cases were matched to 136 test-negative controls with no match found for 129 cases and 24 controls
(matched by CCI category and week of admission).
 ≥50y: 203 test-positive cases were matched to 335 test-negative controls with no match found for 92 cases and 427 controls
(matched by CCI category and week of admission).
≥75y: 90 test-positive cases were matched to 162 test-negative controls with no match found for 20 cases and 371 controls
(matched by CCI category and week of admission).

†All adults: 134 test-positive cases with LOS>3 days were matched to 146 test-positive cases with LOS≤3 days with no match found for 176 cases with LOS>3 and 11 cases with LOS≤3 days
(matched by age). <50y: 82 test-positive cases with LOS>3 days were matched to 93 test-positive cases with LOS≤3 days with no match found for 51 cases with LOS>3 and 9 cases with LOS≤3 days
(matched by age).
≥50y: 52 test-positive cases with LOS>3 days were matched to 53 test-positive cases with LOS≤3 days with no match found for 125 cases with LOS>3 and 2 cases with LOS≤3 days
(matched by age).
≥75y: 14 test-positive cases with LOS>3 days were matched to 15 test-positive cases with LOS≤3 days with no match found for 44 cases with LOS>3 and 2 cases with LOS≤3 days
(matched by age).

# Prevalence was calculated on a daily basis

**Supplementary Table 3:** **Vaccine effectiveness for three-dose BNT162b2 against the Omicron (B.1.1.529) variant**

CI, Confidence Interval; CCI, Charlson comorbidity index; FiO2, Fraction inspired oxygen; IMD, index of multiple deprivation; OR, Odds Ratio; VE, vaccine effectiveness; WHO, World Health Organisation

*All adults: 331 test-positive cases were matched to 529 test-negative controls with no match found for 18 cases and 138 controls (matched by age).
<50y: 66 test-positive cases were matched to 89 test-negative controls with no match found for 14 cases and 47 controls (matched by age).
≥50y: 265 test-positive cases were matched to 440 test-negative controls with no match found for 4 cases and 91 controls (matched by age).
≥75y: 182 test-positive cases were matched to 308 test-negative controls with no match found for 44 controls (matched by age).

⁑ All adults: 52 test-positive cases were matched to 61 test-negative controls with no match found for 297 cases and 606 controls (matched by age, CCI category and week of admission).

†All adults: 69 test-positive cases with LOS>3 days were matched to 87 test-positive cases with LOS≤3 days with no match found for 84 cases with LOS>3 and 45 cases with LOS≤3 days (matched by age).

# All adults: 99 test-positive cases with WHO score>5 were matched to 135 test-positive cases with WHO score ≤5 days with no match found for 33 cases with WHO score>5 and 71 cases with WHO score ≤5 days (matched by age).

## Prevalence was calculated on a daily basis

**Supplementary Table 4: Vaccine effectiveness of BNT162b2 against hospitalisation with Delta (B.1.617.2) and Omicron (B.1.1.529) variant by time since last vaccination**

CI, Confidence Interval; CCI, Charlson comorbidity index; FiO2, Fraction inspired oxygen; IMD, index of multiple deprivation; OR, Odds Ratio; VE, vaccine effectiveness; WHO, World Health Organisation

*All adults: 296 test-positive cases matched to 353 test-negative controls with no match found for 156 cases and 146 controls (matched by admission week).

<50y: 101 test-positive cases were matched to 125 test-negative controls with no match found for 133 cases and 10 controls (matched by week of admission).

≥50y: 145 test-positive cases were matched to 194 test-negative controls with no match found for 73 cases and 170 controls (matched by week of admission).

≥50y: 38 test-positive cases were matched to 59 test-negative controls with no match found for 16 cases and 143 controls (matched by week of admission).

⁑All adults: 110 test-positive cases matched to 133 test-negative controls. No match found for 414 cases & 607 controls (matched by age & admission week).

<50y: 108 test-positive cases were matched to 129 test-negative controls with no match found for 127 cases and 7 controls (matched by week of admission).

≥50y: 222 test-positive cases were matched to 412 test-negative controls with no match found for 67 cases and 192 controls (matched by week of admission).

≥50y: 89 test-positive cases were matched to 164 test-negative controls with no match found for 19 cases and 255 controls (matched by week of admission).

**Supplementary Table 5: Vaccine effectiveness of BNT162b2 against LOS>3days, WHO score>5, FiO2 >28% with Delta (B.1.617.2) and Omicron (B.1.1.529) variant by time since last vaccination**

CI, Confidence Interval; CCI, Charlson comorbidity index; FiO2, Fraction inspired oxygen; IMD, index of multiple deprivation; OR, Odds Ratio; VE, vaccine effectiveness; WHO, World Health Organisation

**Supplementary Table 6:** **Sensitivity analysis -** **Vaccine effectiveness for three-dose BNT162b2 against hospitalisation against the Omicron (B.1.1.529) variant (all adults)**

CI, Confidence Interval; CCI, Charlson comorbidity index; IMD, index of multiple deprivation

Sensitivity analysis on different matching methods and criteria resulted in substantial differences in the study sample and consequently in the outcomes. These results should be interpreted with caution; higher VE estimates from the conditional logistic regression after exact matching are associated with case-control pairs belonging to older age groups with higher CCI, while lower estimates result from matched pairs that are more representative of the initial sample under study, both indicating a selection bias ^2^. *All matching methods were performed using the MatchIt package^1^ in R.

1 Ho D, Imai K, King G, Stuart EA. : Nonparametric for Parametric Causal Inference. *J Stat Softw* 2011; **42**: 1–28.

2 Mansournia MA, Jewell NP, Greenland S. Case–control matching: effects, misconceptions, and recommendations. *Eur J Epidemiol* 2018; **33**: 5–14.

**Supplementary Table 7:** **Sensitivity analysis -** **Vaccine effectiveness for two-dose BNT162b2 against hospitalisation against the Delta (B.1.617.2) variant (all adults) including additional interaction terms between vaccine and age (18–34, 35–49, 50-64, 65-74, 75-84 and 85+ years)**

OR, Odds Ratio; CI, Confidence Interval; CCI, Charlson comorbidity index; IMD, index of multiple deprivation
